# Mesenchymal Stem/Stromal Cell Therapy in Blood–Brain Barrier Preservation Following Ischemia: Molecular Mechanisms and Prospects

**DOI:** 10.3390/ijms221810045

**Published:** 2021-09-17

**Authors:** Phuong Thao Do, Chung-Che Wu, Yung-Hsiao Chiang, Chaur-Jong Hu, Kai-Yun Chen

**Affiliations:** 1 International Ph.D. Program for Cell Therapy and Regeneration Medicine, College of Medicine, Taipei Medical University, Taipei 110, Taiwan; dr.dophuongthao@gmail.com; 2 Department of Pediatrics, Hanoi Medical University, Hanoi 100000, Vietnam; 3 Department of Neurosurgery, Taipei Medical University Hospital, Taipei 110, Taiwan; johnwu@tmu.edu.tw (C.-C.W.); ychiang@tmu.edu.tw (Y.-H.C.); 4 Department of Surgery, School of Medicine, College of Medicine, Taipei Medical University, Taipei 110, Taiwan; 5 TMU Neuroscience Research Center, Taipei Medical University, Taipei 110, Taiwan; 6 Taipei Neuroscience Institute, Taipei Medical University, Taipei 110, Taiwan; 7 Department of Neurology, School of Medicine, College of Medicine, Taipei Medical University, Taipei 110, Taiwan; 8 Department of Neurology and Stroke Center, Shuang Ho Hospital, Taipei Medical University, New Taipei City 235, Taiwan; 9 The PhD Program for Neural Regenerative Medicine, College of Medical Science and Technology, Taipei Medical University, Taipei 110, Taiwan

**Keywords:** blood–brain barrier, permeability, cell therapy, matrix metalloproteinases, inflammation, ischemic stroke, mesenchymal stem cell, molecular mechanism

## Abstract

Ischemic stroke is the leading cause of mortality and long-term disability worldwide. Disruption of the blood–brain barrier (BBB) is a prominent pathophysiological mechanism, responsible for a series of subsequent inflammatory cascades that exacerbate the damage to brain tissue. However, the benefit of recanalization is limited in most patients because of the narrow therapeutic time window. Recently, mesenchymal stem cells (MSCs) have been assessed as excellent candidates for cell-based therapy in cerebral ischemia, including neuroinflammatory alleviation, angiogenesis and neurogenesis promotion through their paracrine actions. In addition, accumulating evidence on how MSC therapy preserves BBB integrity after stroke may open up novel therapeutic targets for treating cerebrovascular diseases. In this review, we focus on the molecular mechanisms of MSC-based therapy in the ischemia-induced prevention of BBB compromise. Currently, therapeutic effects of MSCs for stroke are primarily based on the fundamental pathogenesis of BBB breakdown, such as attenuating leukocyte infiltration, matrix metalloproteinase (MMP) regulation, antioxidant, anti-inflammation, stabilizing morphology and crosstalk between cellular components of the BBB. We also discuss prospective studies to improve the effectiveness of MSC therapy through enhanced migration into defined brain regions of stem cells. Targeted therapy is a promising new direction and is being prioritized for extensive research.

## 1. Introduction

Stroke is the second leading cause of death worldwide [1,2], while the available therapeutic options are limited. Moreover, the pathophysiology of ischemia-reperfusion brain injury exhibits extremely complex vicious cycles, including prominent events such as increased blood–brain barrier (BBB) permeability, infiltration of immune cells, robust inflammatory response, oxidative stress, and apoptosis [3,4]. The BBB is a distinctive structure found only in the brain and plays a vital role in the homeostasis of the central nervous system (CNS) through tight regulation of ion and nutrient transport processes and prevention of neurotoxic molecules from the circulatory system [5,6,7]. The impaired BBB is responsible for secondary brain injuries of the CNS after stroke, traumatic brain injury, epilepsy, and more [8,9]. Mechanisms of ischemia-induced BBB breakdown involve matrix metalloproteinase (MMP) activation, basement membrane degradation, and impaired cell–cell connections of the neurovascular unit [5,10,11]. Clinical and preclinical studies have demonstrated that MMPs are upregulated post-stroke, resulting in the breakdown of tight junction proteins (TJs) [12,13,14]. In addition, the morphological integrity and interactions between cellular components of the brain barrier, such as pericytes, astrocytes, and brain microvascular endothelial cells (BMVECs), play a pivotal role in regulating BBB permeability [15]. With the robust development of science and technology in recent years, the pathogenesis of BBB damage after stroke has gradually been clarified, opening up great potential for targeted treatment strategies.

Currently, the two approved treatments for reperfusion following acute ischemic stroke are recombinant tissue plasminogen activator administration and mechanical thrombectomy [16]. However, the benefits of recanalization treatments have been considerably restricted in most patients due to the strict therapeutic time window and reperfusion injuries such as hemorrhagic transformation [16,17,18]. The vigorous development of preclinical and clinical studies using cell therapy in stroke treatment has emerged worldwide in the past decades, and demonstrated that stem cell therapeutic strategies diminished infarct volume and significantly ameliorated neurological deficits following ischemia [19,20]. Potential mechanisms for this therapy involved angiogenesis and neurogenesis promotion, reduction of apoptosis and neuroinflammation via stem cell secretomes [21,22]. Several types of stem/progenitor cells such as embryonic stem cells [23,24], neural stem cells [25], mesenchymal stem cells (MSCs) [26,27], endothelial progenitor cells [28], and induced pluripotent stem cells [29] have been evaluated as potential cell-based therapy for ischemic brain injury. Among these, MSCs are considered excellent candidates for post-stroke cell therapy due to their advantages, including feasibility (self-renewable, easily accessible, and culturally expandable), potential mechanisms for repairing brain injury, safety in preclinical and clinical practices, and overcoming ethical issues [21,30,31]. MSCs can be isolated from diverse sources such as adipose tissue, bone marrow, umbilical cord, placenta, amnion, dental pulp; these share common characteristics and generally fulfill accepted criteria for MSCs [30,31]. With easy accessibility, MSC-based therapy has been studied extensively in preclinical and clinical trials [32]. Preclinically, MSC transplantation has shown beneficial effects on motor and sensorimotor functions after cerebral infarction in systematic reviews and meta-analysis [32,33]. Results of randomized controlled trials with MSC administration indicated improvement in clinical severity score through the National Institutes of Health Stroke Scale (NIHSS) and modified Rankin scale (mRS) [34].

Numerous subclinical investigations indicated the amelioration of endothelial permeability after MSC treatment with the highly complex relationship of cellular components of BBB and intercellular junctions [27,35,36,37]. Understandably, BBB preservation post-ischemia is receiving significant attention from investigators, promising to explore novel therapeutic targets. Nevertheless, the molecular mechanisms of MSC therapy in preventing BBB compromise after cerebral infarction have not yet been comprehensively reviewed. Therefore, this review highlights the main underlying mechanisms of MSC therapy involving maintenance of BBB integrity following ischemic stroke in preclinical studies. Concomitantly, we also discuss prospective investigations to improve the efficacy of MSC treatment through enhanced migration into particular brain regions of these stem cells.

## 2. Structure of Blood–Brain Barrier

**Figure 1:**
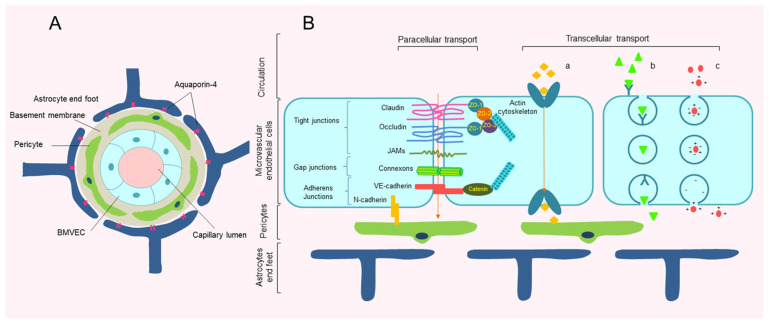
Structure and transportation of metabolites through the blood–brain barrier (BBB). (**A**) Structural component of BBB includes brain microvascular endothelial cells (BMVECs), pericytes, basement membrane, and astrocytic endfeet. (**B**) Mechanism of transport across BBB. Major interendothelial junctions consist of tight junctions, adherens junctions, and gap junctions. Tight junction proteins (TJs) are composed of transmembrane proteins (claudins, occludins) and connecting adhesion molecules (JAM) which contribute to paracellular connection and interaction with zonula occludens (ZO) proteins and actin cytoskeleton. (**a**) Carrier-mediated transport systems (glucose, amino acid exchange); (**b**) specific receptor-mediated endocytosis (insulin exchange); (**c**) adsorptive endocytosis (plasma proteins exchange).

The blood–brain barrier (BBB) comprises a layer of BMVECs tightly held together by intercellular junctions, pericytes embedded in the basement membrane, and astrocytic endfeet [5,38,39] (Figure 1A). The exchange of molecules across BBB is based on two fundamental mechanisms: transendothelial transport and paraendothelial transport. BMVECs have distinctive properties, such as high transendothelial electrical resistance (TEER), rigorously selected transcytosis and paracellular transport, which pivotally contribute to homeostasis maintenance of the CNS [5,40,41]. The major components of the interendothelial cleft include tight and adherens junctions, which play crucial roles in the diffusion of small molecules through the paracellular pathway [5,42]. The BBB controls transcytosis of substrates through a series of distinctive structures, such as specific transporters, ion channels, and energy-dependent pumps [4,43].

Tight junction proteins (TJs) are complex structures with multiple transmembrane proteins, such as claudins, occludins, and junctional adhesion molecules (JAMs), associated with auxiliary cytoplasmic proteins like zonula occludens (ZO) and cytoskeleton-related proteins [39,44]. Evidence suggests that occludins phosphorylation causes increased BBB permeability [45], whereas claudins contribute to the selective opening of TJs [46]. Furthermore, the main interendothelial adhesive junction is the VE-cadherin protein, which anchors to the actin cytoskeleton via catenins, participates in cohesion and maintenance of BMVEC integrity as well as proper vascular development [47,48]. Therefore, TJs and adherens junctions together establish a stable endothelial layer. The gap junctions are intercellular hemichannel pairs (or connexons) that directly connect the cytoplasm of adjacent cells [49]. Connexin 43 is the most abundant gap junction protein in the brain, and participates in the propagation of electrical and chemical signals between astrocytes and adjacent cells [50].

The smooth coordination of cellular components, such as BMVECs, astrocytes, and pericytes, pivotally contributes to BBB consistency [47]. Astrocytes are the most common glial cells in brain that directly connect to BMVECs through their endfeet [51]. The morphological enclosing of the entire cerebral microvascular system, by the terminal feet of astrocytes and their interactions with BMVECs through extracellular vesicles, determines the BBB properties [51,52,53]. In addition, astrocytes contribute to the stability of TJs through VEGF-mediated signal transduction [27,36], and regulating tissue inhibitors of metalloproteinases (TIMPs) [54].

Brain vascular pericytes are multipotent cells embedded in the basement membrane and enclose microvessels [55,56,57,58]. Pericyte–endothelial interaction is critical in paracellular permeability, profoundly affecting the basement membrane, and TJ structure and function [59]. Previous studies have revealed that pericytes stimulate the production of TJs, particularly occludins, claudin-1, ZO-1, and ZO-2, by secreting pro-angiogenic factors [59,60]. Moreover, pericytes support the function of BMVECs and regulation of cerebral blood flow by conducting signals in gap and adherens junctions [59,61]. In addition, pericytes simultaneously assist in the elongation and polarization of astrocytic endfeet toward the microvascular wall, facilitating the maturation of the BBB [62].

## 3. Blood–Brain Barrier Changes Following Ischemia-Induced Brain Injuries

### 3.1. Tight Junction Disruption and Blood–Brain Barrier Opening

The disturbance of blood–brain barrier (BBB) function after stroke varies depending on the mechanism, severity, and duration of ischemia [9]. Ischemia-reperfusion injury may induce a biphasic opening of the brain barrier, triggering a series of secondary lesions. The early stage of BBB opening takes place within several hours after ischemia and may reverse, involving the effect of MMP-2 in loosening TJs [9,63,64]. In contrast, the second stage starts from 24 to 72 h following ischemia-reperfusion insult and is irrecoverable with the significant contribution of MMP-9 [12,63]. Otherwise stated, the early stage corresponds to TJs impairment; the late barrier opening may be involved in neuroinflammation and cell destruction [9,63].

Hypoxic conditions can result in BMVEC injury due to the uncontrolled generation of free radicals, pro-inflammatory factors, including tumor necrosis factor (TNF-α), interleukin-1 (IL-1), adhesion molecules, and chemo-attractants (Chemokine (C-C motif) ligand 19-CCL19, stromal cell-derived factor-1) [4,65]. Therefore, reactive leukocytes are attracted to the injured site and penetrate through the disrupted endothelium. Neutrophils are the most dominant immune cells in the acute post-stroke period [66,67]. Neutrophils undergo sequential rolling, adhesion, and transendothelial infiltration into the brain parenchyma, consequently stimulating the release of reactive oxygen species (ROS), pro-inflammatory factors, and proteases, leading to the increase of vascular permeability, cytotoxicity and vasogenic edema [4,5,9].

**Figure 2:**
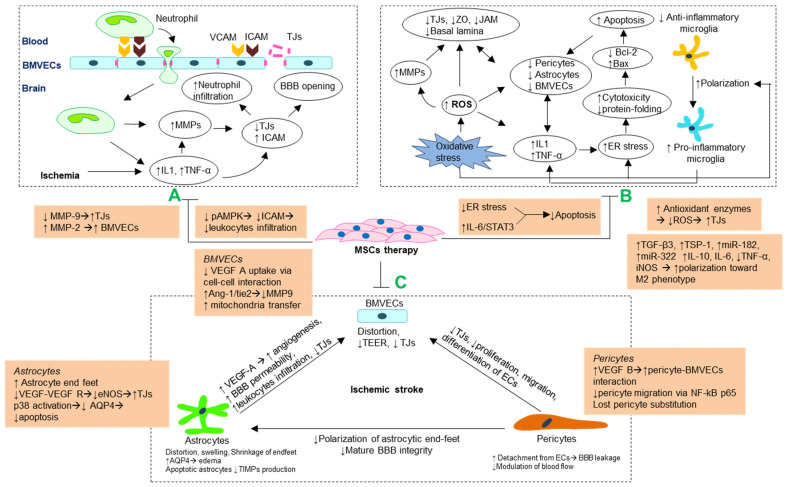
Schematic illustration of the major molecular mechanisms of ischemia-induced BBB disruption. (**A**) MMP regulation and attenuating leukocytes infiltrations. (**B**) Antioxidant and anti-inflammatory mechanism (comprising antioxidants, polarization toward anti-inflammatory macrophage and anti-apoptosis). (**C**) Stabilizing morphology and crosstalk of cellular components in BBB.

Neutrophils not only secrete on their own but also activate the release of MMPs from resident cells [67,68]. MMPs are zinc-containing proteases that can break down the basement membrane and extracellular matrix [11,64,69]. In particular, MMP-9 or gelatinase B is a collagenase that can disintegrate TJs and basal lamina proteins, thereby leading to brain barrier disruption [64]. Transmigrated leukocytes are the primary source of MMP-9, causing BBB interruption following stroke through increasing transcriptional activity and activation of pre-MMP-9 by N-nitrosylation and oxidation [63,68]. Ischemic-induced MMP-9 upregulation is closely related to irreversible BBB opening, neutrophil extravasation, vasogenic edema, and hemorrhage [70,71]. Consequently, downregulating leukocyte recruitment and MMP-9 could be a promising strategy to ameliorate BBB leakage after stroke (Figure 2).

### 3.2. Morphological Changes and Impaired Interactions of Cellular Components of Blood–Brain Barrier

Pericytes, astrocytes, and BMVECs tightly communicate together by either direct structural contact or indirect secretomes. Therefore, morphological or functional disturbance of the cellular components of BBB in hypoxic conditions may be responsible for cerebrovascular leakage [15] (Figure 2). Electron micrographs confirmed morphological changes in the BBB, including distortion and swell of BMVEC nuclei, mitochondrial loss, and edema of the astrocyte endfeet at initial hours after reperfusion, and further irregular endothelium surface, vacuolation, and enlargement of perivascular spaces due to severe destruction of astrocyte endfeet [72]. Indeed, astrocytes responded to hypoxia by contracting their endfeet from the capillaries, caused disruption of BBB structure and increased vascular permeability [72,73]. In particular, ischemia-induced astrocyte dysfunction can directly affect the endothelium by promoting vascular or cellular edema of aquaporin-4 (AQP4) [74], increasing TJ and basement membrane degradation factors (MMPs) [51,75], and releasing pro-inflammatory factors (IL-1β, TNF-α) [76]. AQP4 is a water channel protein, resides on the endfeet of astrocytes. This transport system increases water entry during ischemia, eventually leading to swollen and apoptotic astrocytes [77]. Moreover, astrocytes also release vascular endothelial growth factor A (VEGF-A), resulting in leukocyte infiltration through degradation of TJs [73,78]. The combination of VEGF-A and its receptor on BMVECs activates endothelial NO synthase (eNOS), deteriorates TJs, and further enhances barrier disruption [52]. In addition to negative responses, astrocytes are involved in numerous beneficial processes, including angiogenesis promotion [27], immune modulation and neuroprotection [79], brain barrier regulation, and axon regeneration [80]. Consequently, astrocyte-targeted therapies could be a useful solution for post-stroke regeneration.

Pericytes regulate various critical processes following stroke, including cerebrovascular permeability, cerebral blood flow, and repair of BBB components [57]. During ischemia, granular pericytes separate from BMVECs, leading to BBB leakage through TJ degradation and enhanced paracellular transport [81]. These pericytes act as repair and scavenger cells. Meanwhile, filamentous pericytes are involved in the adjustment of the capillary lumen size. Although shrinkage of pericytes is beneficial in providing sufficient oxygen to neurons in a healthy brain, this also aggravates hypoxia following stroke [57]. Furthermore, pericytes may contribute to the proliferation and maturation of BMVECs through the expression of VEGF [82], platelet-derived growth factor receptor (PDGFR) [83], transforming growth factor- (TGF-) [84], and stabilizing actin filaments [5]. Pericyte detachment from BMVECs induces basement membrane rupture through upregulation of MMPs, facilitates vascular budding after stroke [55]. Together with tubulogenesis, pericytes also activate the function of transporters, localize barrier proteins, and polarize the lumen during the remodeling phase [85].

### 3.3. Increase of Blood–Brain Barrier Permeability Following Vascular Remodeling

The angiogenesis process initiates from 12 to 24 h following the ischemic attack, leading to increased microvascular density in peri-infarcted areas [86,87]. Following primary angiogenesis, newborn vessels undergo a barriergenesis process, characterized by the migration of pericytes and astrocyte endfeet, forming the extracellular matrix, eventually establishing a complete BBB [85,88]. Enhanced angiogenesis helps restore oxygen and nutrient supply, subsequently improving neurological outcomes [89]. Unfortunately, some angiogenic therapy, like VEGF treatment, can induce TJ disruption and BBB leakage. In other words, VEGF can promote BMVEC proliferation but not maturation [78,90]. MRI and histological techniques effectively evaluate the morphological and functional new capillaries formed. The central area of the infarction has a severe decrease in cerebral blood flow; in the opposite direction, abundant perfusion occurs in the penumbra (peri-infarct) region observed by the arterial spin labeling technique [91]. Furthermore, evidence indicated that leakage of the penumbra areas remarkably increased by enhancing plasma volume (Vp) and BBB transfer rate (Ki) in the permeability coefficient maps [92]. Correspondingly, immunohistochemical staining for an endothelial marker (RECA-1) revealed increased signals surrounding ischemic regions, almost absent in the core area [93]. These findings suggest that either new angiogenic vessel processes or increased BBB permeability occur in the peri-infarct regions.

## 4. Potential Mechanisms of Blood–Brain Barrier Preservation by MSCs Following Ischemia

### 4.1. MMP Regulation and Attenuating Leukocytes Infiltrations

Under ischemic conditions, transmigrated neutrophils secrete MMPs that damage TJs, amplify vascular permeability, and initiate neuroinflammatory cascades, which may cause cerebral edema and severe neurological deficits [4,65]. MMP-9 is significantly upgraded in the late phase of stroke and can lead to irreversible BBB disruption [12,13]. Evidence indicated that MMP9 activity was considerably downregulated by MSC transplantation, whereas MMP2 activity was unaltered [68,94]. Therefore, inhibition of MMP-9 is an effective targeted therapy for preventing BBB compromise. Evidence suggests that MSCs can attenuate MMP-9 upregulation from extravasated neutrophils and resident cells, contributing to BBB preservation, reducing infarct volume and neurological deficits following ischemic stroke [68,69,89,95]. Cheng et al. reported that MSC transplantation remarkably reduced IgG leakage through declining MMP-9, TNF-α, and pro-inflammatory factors (IL-1β, IL-6) expression, and neutrophil penetration in transient middle cerebral artery occlusion (MCAO) models [68]. Using anti-Ly6G delivery to induce neutrophil depletion, Wang and colleagues proved that MSC-derived extracellular vesicles treatment was ineffective in decreasing brain damage and infiltration of other types of immune cells such as monocyte/macrophage and lymphocyte [67]. Alternatively stated, blocking neutrophil penetration is one of the key mechanisms of MSCs therapy [67,68] (Figure 2).

Intercellular adhesion molecules 1 (ICAM-1) is a ligand for leukocyte’s integrin, directly involved in the transmigration of these immune cells [96]. MSCs suppress ICAM-1 expression through AMP-activated protein kinase (AMPK) [68]. AMPK refers to a heterotrimeric kinase that controls the cellular enzymes, acting as an energy sensor to maintain metabolic homeostasis and synthesis [97]. Upregulation of ICAM and phosphorylation of AMPK in BMVECs increased following ischemia but reversed after co-culturing with MSCs. These effects might be mediated via an AMPK-dependent ICAM-1 downregulation in BMVECs, consequently impairing leukocyte extravasation and preventing BBB compromise. Collectively, ICAM-1 might be a pivotal paracrine factor of MSCs in regulating leukocytes diapedesis [68] (Table 1). Nevertheless, elevated ICAM expression is proportional to immunosuppressive capacity of MSCs [98], promotes adhesion of MSCs to endothelial cells through p38 mitogen-activated protein kinases (MAPKs) signaling pathway [99]. Therefore, the regulation of ICAM by MSCs needs to be further investigated to optimize the benefits of stem cell therapy.

Tissue inhibitors of metalloproteinases (TIMPs) are endogenous proteins that neutralize MMPs, stabilize the extracellular matrix, and reduce BBB disruption following stroke [11,100]. Indeed, more severe BBB interruption and neuronal apoptosis were reported in cerebral ischemia-induced TIMP-1 knockout mice [101]. Correspondingly, BBB leakage and infarction volume significantly improved in TIMP-1 overexpressed mice following ischemic injury [102]. TIMP-1 can inhibit a wide range of MMPs, even though it has been described as particularly potent against MMP-9 [103]. TIMP3 expression after MSCs administration considerably alleviated BBB permeability through blocking VEGF-A-induced breakdown of interendothelial junctions [104]. Nevertheless, Bharath et al. revealed that MSC-mediated downregulation of MMPs after stroke was not induced via the TIMP pathway. Ischemia-induced injuries promoted upregulation of all MMPs (MMP-7, -8, -9, -11, -12, -14, -21, and -28) and all four TIMPs (TIMP-1, -2, -3, and -4); subsequently attenuated by MSC therapy [14]. This inconsistency is probably related to the different roles of MMPs and TIMPs that need to be studied further in the future.

Although an increase of MMPs in the initial stage seriously degrades TJs and damages the BBB, MMPs are considerably impaired in the late stages, affecting regeneration and glial scarring formation [105]. Indeed, evidence indicates that TIMP-1 and TIMP-2 simultaneously upregulate MMP-9 and MMP-2 in astrocytes and leukocytes. Therefore, TIMPs might regulate MMPs through not only inhibition but also activation during post-traumatic neurological regeneration [80]. Moreover, other evidence suggested that upregulation of MMP-9 facilitated MSCs migration into targeted areas [106], whereas elevated MMP-2 level may promote proliferation and maturation of endothelial cells [89], further improving neurological outcomes after stem cell administration. MSCs may regulate MMPs in distinct ways concerning different stages after stroke. Therefore, further investigations to elucidate these mechanisms need to be continued in the future.

### 4.2. Antioxidant and Anti-Inflammatory Mechanism

Proinflammatory and reactive oxygen species (ROS) secreted by immune cells such as neutrophils and monocytes/macrophages are considered central factors of TJ disruption and BBB leakage following oxidative stress-induced injuries [107]. Therefore, the antioxidant effects of MSC therapy, reported in various investigations, might be a potential strategy for endothelium barrier restoration. Evidence showed that CCR2-overexpressed MSCs (MSC^CCR2^) preserve BBB integrity by alleviating ROS production and TJ breakdown in vivo, involving the role of CCR2 in MSC homing enhancement. Meanwhile, co-culturing BMVECs with MSC^CCR2^/MSC^control^ reduced OGD-activated TJ loss and ROS levels in vitro, suggesting an antioxidant mechanism of MSC secretomes. Based on genome-wide RNA sequencing (RNA-seq) analysis, a series of antioxidant-related genes of MSCs were screened, revealing high expression of the peroxiredoxin (PRDX) antioxidant enzyme family. Among these peroxiredoxins, PRDX4 dominantly contributes to antioxidant-mediated BBB preservation. Using short interfering RNA against PRDX4 (shPRDX4) to block the effect of PRDX4 impairs the antioxidant effects of MSC^CCR2^, leading to an increase in BBB leakage [108]. Briefly, the PRDX4-mediated antioxidant pathway might be a potential mechanism of MSCs in preventing microvascular barrier disruption. In addition, MSC therapy also enhanced the secretion of other antioxidant enzymes such as heme oxygenase-1 (HO-1) through the Cx43/Nrf2 signaling pathway, significantly reducing brain edema and cell death [109].

The anti-inflammatory actions of MSCs are characterized by the downregulation of pro-inflammatory cytokines [110], prevention of leukocyte penetration [95], and promotion of polarization toward the M2 phenotype of microglia [94] (Figure 2). Cheng et al. transplanted MSCs into stroke mice via the intracerebral ventricular route and reported simultaneous reductions of IL-1β and TNF-α, neutrophil recruitment, and BBB leakage in the treated group [68]. The neuroinflammatory response and increased BBB permeability establish a vicious cycle that is difficult to control; thus, inflammatory inhibition can stabilize BBB function and vice versa [110]. Indeed, the investigation of Yoshida et al. exhibited that the BBB integrity significantly increased in the human amniotic mesenchymal stem cells (hAMSC) injected group, accompanied by a decrease of TNF-α and iNOS, and suppression of microglial transformation towards the pro-inflammatory M1 phenotype [94]. Amnion stem cell-induced M2 macrophage polarization and further secretion of anti-inflammatory cytokines (IL-10 and IL-6) might contribute to repairing the injured brain areas [111]. In addition, MSC secretomes, including tissue growth factor-β3 (TGF-β3), thrombospondin-1 (TSP-1) [112], miR-182 [113], and miR-322 [114] also contribute to the polarization toward anti-inflammatory macrophage direction.

Another beneficial effect of MSCs on BBB maintenance involves the anti-apoptosis of astrocytes [110,115,116] through suppression of endoplasmic reticulum (ER) stress [110], IL-6/STAT3 signaling pathway [115], and Cx43/Nrf2 interaction [109]. Evidence has revealed that markers for pro-apoptotic processes like Bax were downregulated, whereas the expression of anti-apoptotic proteins as Bcl-2 was upregulated after transplantation of MSCs [110,115]. ER stress causes impairment in folding proteins such as GRP78, XBP-1 PERK, eIF2a, ATF4, CHOP, and cytotoxicity, consequently leading to apoptosis [117,118]. Chi and colleagues confirmed that blocking ER stress of MSCs induced suppression of the pro-apoptotic pathway more dominant than anti-apoptotic promotion [110]. In addition, IL-6 is a crucial factor for astrogliosis and BBB consistency, which could be upregulated following cerebral infarction [119]. Oxygen-glucose deprivation (OGD) alleviated IL-6 levels in astrocytes, while co-culture with MSCs remarkably improved IL-6 secretion. Concomitantly, the anti-apoptotic mechanism of IL-6 in astrocytes might be directly involved in the IL-6/STAT3 signaling pathway [115]. Moreover, MSC-based therapy also enhances connexin 43 (Cx43) and nuclear factor erythroid 2-related factor 2 (Nrf2) expression, promotes antioxidant reactions of astrocytes, including increased secretion of heme oxygenase-1 (HO-1) enzyme and impaired apoptosis [109].

### 4.3. Stabilizing Morphology and Crosstalk of Cellular Components Blood–Brain Barrier

#### 4.3.1. Brain Microvascular Endothelial Cells

The morphological and functional stability of BMVECs is vital for ensuring BBB consistency. Indeed, intravenous administration of human adipose-derived MSCs dramatically improved disruption, engorgement, and distortion of the microvasculature, thus reducing BBB leakage in stroke rats [95]. Annexin A1 (ANXA1) is expressed in microglia and BMVECs [120,121] and acts as an anti-inflammatory agent through an agonist of formyl peptide receptors (FPRs) [122,123]. Furthermore, ANXA1 is found in the extracellular vesicles of MSCs [124,125]. Gussenhoven et al. evaluated endothelial resistance through TEER values following oxygen-glucose deprivation (OGD) induction. MSC-derived extracellular vesicle (MSC-EV) treatment gradually improved TEER values and stabilized them at 122 Ω, 12 h after OGD; however, this amelioration was not observed after inhibiting FPR1 and FPR2 receptors [125]. In addition, BBB leakage occurs in ANXA1 knockout mice due to endothelial TJ degradation and actin microfilament instability [126]. ANXA1-FPR2 receptor interaction can inactivate the small GTPase RhoA, linking β-actin to the plasma membrane and facilitating TJ formation [126,127]. On the other hand, ANXA1 induces phagocytosis of apoptotic cells and debris by microglia without eliciting a pro-inflammatory response [128], and promotes microglial polarization and migration [121]. Therefore, the molecular mechanism of BBB preservation of MSCs might involve endothelium layer stabilization and anti-inflammatory effect through the ANXA1/FPR-axis.

MSCs exhibit another potential mechanism involving the suppression of VEGF-induced BBB leakage. Kikuchi-Taura and colleagues suggested that MSCs uptake glucose from endothelial cells and inhibited the absorbance of VEGF into these cells, thus reducing BBB permeability. Immunohistochemistry image showed the overlap of connexin (Cx) 37, 43, MSCs and BMVECs signals. Moreover, blocking the gap junction channel of MSCs reversed the VEGF uptake of BMVECs in vitro and in vivo. These results revealed cell–cell interaction between MSC and BMVECs through gap junction [36]. Previous studies have shown that VEGF stimulates angiogenesis and increases BBB permeability, promoting inflammatory responses following stroke [129,130]. Concomitantly, MSCs and circulating lymphocytes/monocytes also established a direct interaction via gap junction. In brief, gap junction-mediated MSC-recipient cell interaction serves as a potential therapy to warrant BBB steadiness after ischemic brain injury involving suppression of VEGF uptake and inflammatory responses [36].

Recently, endothelial mitochondria have played an essential role in cellular responses to environmental stresses, notably oxidative stress, which profoundly affected the BBB integrity [131]. Tunneling nanotubes (TNTs) is a distinct method of intercellular communication involving the transport of organelles such as mitochondria, intercellular vesicles, lysosomes, lipid droplets, viral genome, and so forth [132]. MSCs transferred healthy mitochondria to damaged endothelial cells via TNTs, contributing to the restoration of hypoxia-induced vascular injuries in both in vitro [133] and in vivo [134]. Oxygen glucose deprivation/reoxygenation (OGD/RO) stress stimulated TNTs generation via membrane protrusions and surface-exposed phosphatidylserines, promoted a mostly unidirectional mitochondrial transport from MSCs to endothelial cells, concomitantly removed damaged mitochondria by lysosomal transfer, thus significantly diminished anaerobic metabolism and apoptosis processes of vascular cells [133]. Transplantation of MSCs into the rat MCAO model established a significant correlation between microvessel density and the number of transferred mitochondria from MSCs [134]. Moreover, evidence revealed that Cx43 [135] and Rho-GTPase 1 [136] could enhance MSC-mediated mitochondria transfer.

#### 4.3.2. Astrocytes

MSCs can enhance the interaction between astrocytes and BMVECs in regulating angiogenesis and vascular maturation (Table 1 and Figure 2). Although VEGF serves as a vascular permeability factor in the initial stages of a stroke, it also plays an essential role in BMVEC proliferation and survival [35,90]. VEGF combines with its receptor on BMVECs, VEGF receptor 2 (Flk1) and directly participates in angiogenic promotion [137]. Whereas, angiopoietin-1 (Ang-1)–Tie-2 interaction recruits mural cells to wrap around BMVECs, thus contributes to maturation and stabilization of new capillaries [138]. Angiogenesis-induced BBB leakage after brain injury significantly reduced through enhancing the release of Ang-1 from astrocytes [139,140]. Indeed, MSC therapy promoted astrocyte-BMVECs crosstalk via endogenous Ang1 and VEGF upregulation and their respective receptors Flk1 and Tie2 in BMVECs, thus increased endothelial occludins expression and BBB integrity. In addition, inhibition of Flk1, Ang1, and Tie2 attenuated remarkably MSC-activated capillary tube formation. VEGF/Flk1 and Ang1/Tie2 systems might be involved in the beneficial effect of MSC therapy in BBB stabilization following ischemia [35,141]. In addition, Ang-1 diminished MMP-9 activity, thereby improved VEGF-induced endothelial barrier leakage [90]. Thrombospondin-4 (TSP4)-overexpressing MSC increased all VEGF, Ang-1, MMP-9, MMP-2 expression through the TGF-β/Smad2/3 signaling pathway and improved endothelial proliferation and migration [142].

MSCs affect BBB consistency based on either astrocytic morphology integrity or astrocyte-secreted angiogenic factors [27,35]. MSCs can restore the density of filaments within astrocytic endfeet surrounding microvessels, representing a morphologically stabilizing effect of MSCs on ensuring BBB function [27]. Indeed, MSC transplantation after inducing LPS significantly enhanced the density of astrocytic endfeet around vessels, prevented neutrophil infiltration, and LPS-induced VEGF-A downregulation in astrocytes. To identify the regular mechanism of MSCs on astrocyte-secreted VEGF-A, BMVECs were treated with VEGF-A and then co-cultured with MSCs. MSC treatment induced decrease of VEGF-A level and TJ breakdown via enhancing endothelial nitric oxide synthase (eNOS) expression [27,52,78]. IL-1β secreted by responsive microglia following brain injury initiates the VEGF-A-related eNOS-dependent signaling pathway by interacting with its receptors in astrocytes [27,52,143]. Furthermore, MSCs can considerably promote secretion of the anti-inflammatory cytokines IL-6, IL-10, and TGFβ in astrocytes, thus attenuating microglial activation [27,144]. Collectively, MSC transplantation prevented BBB compromise by preserving astrocytic endfeet around vessels, decreasing LPS-stimulated VEGF-A, and improving eNOS-dependent TJ impairment.

Aquaporin-4 (AQP4) plays an essential role in water homeostasis of CNS [145]. Ischemic brain injury activates AQP4 upregulation, induces astrocyte swell, further increases apoptotic astrocytes and BBB dysfunction [77,145]. Tang et al. showed that MSC treatment inhibited neuroinflammatory factors, consequently suppressing robust AQP4 expression and apoptosis of astrocytes in a transient middle cerebral artery occlusion (tMCAO) model. Knockdown of AQP4 attenuated the apoptosis of cultured astrocytes in vitro via the p38 signaling pathway, but not the JNK pathway. Although p38 and JNK activation occurred in ischemia-induced astrocytes, the study of Tang et al. proved that increase of AQP4 expression is only related to the p38 pathway. This result suggested that p38 might be a dominant pathway of regulation of AQP4 after ischemic stroke. The immunomodulatory functions could explain the beneficial effects of MSCs on AQP4 downregulation in astrocytes via decrease of inflammatory cytokines IL-1β, IL6, and TNF-α [146].

#### 4.3.3. Pericytes

Evidence suggests that MSCs can regulate pericyte morphology and pericyte–BMVEC interaction. Lu and colleagues revealed that cleavage of pericytes from the vessel wall after spinal cord injury (SCI) involves the NF-κB p65 signaling pathway, consequently causing severe BBB breakdown. MSC-derived extracellular vesicle treatment prevented detachment of pericyte from microvascular system through NF-κB p65 pathway inactivation [37]. Furthermore, MSC-extracted growth factors such as VEGF B reinforced the interaction between pericytes and BMVECs and increased pericyte survival, thus considerably improving BBB leakage [147]. Moreover, pericytes are a type of MSCs that serve as pivotal factors in maintaining BBB integrity. Investigations on the BBB mimicking model in vitro showed that MSCs contributed similarly to pericytes in increasing trans-endothelial electric resistance (TEER) and decreasing permeability against macromolecules [148]. Taken together, these findings suggest that MSCs might be used as a potential therapy for BBB preservation by substituting lost pericytes [149].

**Table 1:** Investigation of the molecular mechanism of MSC therapy in blood–brain barrier preservation after ischemic brain injury.

Reference	Signaling Pathway	Component of BBB	Molecular Mechanism	Model	Number of Cells and Sources	Route	Time Treatment/Passage
[68]	ICAM/AMPK	MMPs, ICAM-1	↓ICAM-1 ↓neutrophil infiltration, ↓MMP-9	tMCAO	2 × 10^5^ BMMSC	ICV	15 min/3
[146]	P38	AQP-4 astrocytes	↓AQP-4, ↓neuroinflammatory, ↓apoptotic astrocytes	tMCAO	2 × 10^5^ BMMSC	ICV	20 min/3
[27]	VEGF/eNOS	Astrocytes endfeet	↑density of astrocytic endfeet, ↑VEGF/eNOS-dependent TJs	LPS	1 × 10^6^ BMMSC	IV	4 h/6
[115]	IL-6/STAT3	Astrocytes	↑IL-6 ↑anti-apoptosis of astrocytes	HIBD	2 × 10^5^ BMMSC	ICV	5 days/3–5
[35]	VEGF/Flk1 Ang1/Tie2	Astrocytes BMVECs	↑Ang1/Tie2 → ↑occludins and VEGF/Flk1 expression ↑vascular maturation	tMCAO	3 × 10^6^ BMMSC	IV	24 h/-
[108]	PRDX4	BMVECs	↑PRDX4-mediated antioxidant ↓ ROS	tMCAO	2 × 10^6^ BMMSC	IV	24 h/5–10
[95]	-	BMVECs	↓Neutrophil infiltration ↓Endothelial damage	GCI	1 × 10^6^ ADMSC	IV	Immediately/2
[125]	ANXA1-FPR	BMVECs	↓endothelial resistance	UCO OGD	BMMSC-EVs 2doses ~2 × 10^7^	IV	1,4 days/-
[134]	Mitochondrial TNTs	BMVECs	Transfer mitochondrial to BMVECs via TNTs→↓oxidative stress	tMCAO	5 × 10^5^ BMMSC	IA	24 h/3–5
[133]	Mitochondrial TNTs	hUVECs	Transfer mitochondrial to hUVECs via TNTs→↓oxidative stress	OGD RO	-	-	4 h/3–5
[142]	TGF-β Smad2/3	BMVECs	↑VEGF, ↑Ang-1	pMCAO	2 × 10^6^ BMMSC	IV	3 h/3
[36]	VEGF	Gap junctionBMVECs	↑gap junction-mediated cell-cell interaction ↓glucose, ↓VEGF uptake in ECs	pMCAO	5 × 10^5^ BMMSC	IV	24 h/9
[37]	NF-kB p65	Pericytes	↓NF-kB p65→↓pericyte migration	SCI	BMMSC-EVs 1 dose ~2 × 10^6^	IV	30 min/3–5
[110]	ER stress	ER Astrocytes	↓ER stress-induced apoptosis ↓inflammation	tMCAO	2 × 10^6^ 3 doses ADMSC	IV	0, 12, 24 h/2
[94]	-	-	↓pro-inflammatory, ↓polarize towards M1-phenotype	pMCAO	4 × 10^6^ cells/kg hAMSC	IV	24 h/-

GCI, global cerebral ischemia; tUCO, transient umbilical cord occlusion; tMCAO, transient middle cerebral artery occlusion; pMCAO, permanent middle cerebral artery occlusion; HIBD, hypoxic-ischemic brain damage; OGD, oxygen glucose deprivation; RO, reoxygenation; LPS, lipopolysaccharide; SCI, spinal cord injury; BMMSC, bone marrow stem cell; hAMSC, human amniotic mesenchymal stem cells; ADMSC, adipose-derived mesenchymal stem cells; ER, endoplasmic reticulum stress; BMVECs, brain microvascular endothelial cells; EVs, extracellular vesicles; ICV, intracerebral ventricular; IV, intravenous; IA, intra-arterial; TNTs, tunneling nanotubes.

## 5. Prospective of MSC-Based Strategies

### 5.1. Mesenchymal Stem Cell Therapy

With the explosive development of basic research in recent years, the molecular pathogenesis of cerebral ischemia has gradually been elucidated, suggesting novel targeted strategies. Increasing studies have demonstrated that systemically administered MSCs can migrate into infarct areas, contribute to neurological regeneration through their paracrine actions and differentiation into neuron-like cells or astrocytes [150,151,152]. Although tropism towards the injured brain is a critical property in the repair mechanism of MSCs, overcoming the BBB remains a significant challenge to cell therapy [43,72]. To enhance the extravasation of MSCs through the barrier, many potential strategies have been investigated, including BBB opening (ultrasound, mannitol) [153,154] and MSC modification (genetic modification, drug combination, preconditioning) [155].

Recently, the use of ultrasound (US)-responsive biomaterials comprising microbubbles, liposomes, and nanoparticles have been considered as a prospective theranostic method. Ultrasound-based techniques exert the energy of stimulated microbubbles to induce transient BBB opening and facilitate substrates transport through the brain endothelium [156]. Both focused ultrasound and low-intensity ultrasound combined with microbubbles have been investigated to enhance the transportation of drugs and cells through the BBB [157,158]. Evidence suggests that low-intensity ultrasound-targeted microbubble destruction (LI-UTMD) is probably a non-invasive and effective BBB opening method for MSCs implanted into infarcted brain areas [153,159]. Indeed, Cui et al. reported that the hippocampal BBB was opened via transtemporal ultrasound irradiation, corresponding to ischemic injuries. MSC homing initiated on day 1, progressively elevated, and maximized on day 14 [153]. The cavitation effect activated by the low-intensity ultrasound-irradiated microbubbles stimulated the consecutive migration of MSCs into the cerebral infarct region. Moreover, MRI-guided LI-UTMD allows targeted BBB disruption and assists drug and cell delivery to determined locations in the brain [160,161]. LI-UTMD-supported MSC transmigration remarkably ameliorated neurological outcomes after 1–2 weeks of transplantation in stroke models [153,162]. Furthermore, low-intensity diagnostic ultrasound can be widely applied in clinical practice because of its safety and commercialization. However, the difficulty in controlling the sonication volume and exposure conditions due to the unfocused ultrasound beam limits the effectiveness of this apparatus [153]. Further studies should focus on the exposure window, and the detection of proper pressure amplitude at the targeted site of the brain due to the significant differences in skull thickness, brain size, and acoustic attenuation between humans and animals before clinical application.

Osmotic BBB opening via infusion of a hypertonic solution such as mannitol, fructose, milk amide, and glycerol is a common modality used in preclinical studies to improve drug and cell delivery into the brain parenchyma [154,163]. Osmotic BBB disruption is explained by vasodilatation, dehydration, and shrinkage of BMVECs, leading to the extension of the interendothelial cleft along with the BBB [164]. Barrier impairment facilitates the penetration of stem cells into targeted brain areas, thus enhancing the efficacy of cell therapy. Recent investigations have demonstrated that although mannitol pretreatment did not notably increase the number of cells reaching the lesion sites, enhanced transfer of neurotrophic factors released by stem cells via BBB correlated positively with amelioration of neurological deficits and infarct size [165,166]. The low rate of cellular transmigration may be related to the limited number of intravenously administered cells due to entrapment in pulmonary, liver, spleen capillaries [167], and large-sized stem cells [168]. Moreover, the inconsistency and non-selectivity of BBB opening zones following intra-arterial (IA) mannitol infusion has limited the clinical application of this approach [169]. Intra-arterial injection of mannitol usually influences deep brain regions and rarely affects the cerebral cortex due to the role of local collateral circulation [170], thus, temporary contralateral common carotid artery ligation can considerably increase BBB permeability in the ipsilateral cortex [163]. Recently, real-time MRI has provided a great approach and spatial control for selective BBB opening, applied in investigating BBB interruption by inducing mannitol [171,172] and focused ultrasound [160]. IA infusion and selective BBB opening through real-time MRI can supply localized intervention and minimize systemic exposure [163]. The combination of induced pluripotent stem cells IPSC-derived 3D BBB and osmotic opening of mannitol has opened up new potential research directions involving the transport of agents and cells via the brain barrier in vitro [173]. Briefly, osmotic disruption via mannitol might be an effective approach for MSC delivery into targeted brain areas. However, the side effects of aggravating local inflammatory reactions and the duration of mannitol administration, implanted routes need to be further evaluated.

Genetically modified MSCs offer a broad prospect for the treatment of cerebral infarction. Gene-transfected MSCs either preserve their original therapeutic effects or enhance the benefits of implanted exogenous genes [155]. Enhanced MSC transmigration toward the injured site involves overexpression of some exogenous genes, including FGF21 [174], CCR2 [108] CXCR5 [175], and CXCR4 [106]. However, the potential tumorigenicity and ethical issues associated with transplantation of transgenic cells limit the clinical applicability of this therapeutic approach. In addition, MSC-drug conjunction is a feasible and potential strategy that promotes survival, proliferation, and homing of transplanted cells. In particular, MSC migration into infarcted areas was significantly enhanced in combination with sodium ferulate [176] or in pre-treatment with valproate or lithium [177]; preconditioning refers to changes in physical medium such as hypoxia [178], hyperbaric oxygen [179]. The penetration toward the injured position of hypoxia-preconditioning-induced MSCs increases significantly through upregulation of CXCR4 and MMP-2 [178]. Meanwhile, the long-term hyperbaric oxygen approach can enhance the migration of MSCs or neurogenesis [180]. Collectively, enhancing MSC migration induces angiogenesis, neurogenesis promotion and effectively improves neurological deficits following brain injuries.

### 5.2. MSC-Derived Extracellular Vesicle Therapy

Overcoming the BBB of MSC-derived extracellular vesicles (MSC-EVs) also contributes to the efficacy of stem cell therapy. Improvement in neurological deficits following cerebral insults might be dominantly involved in the paracrine activities of MSCs via EVs [181]. Although EVs are classified into four types, including exosomes, microvesicles (MVs), apoptotic bodies, and oncosomes, only exosomes and MVs play pivotal roles in the therapeutic effects of EV-based strategies. MSC-EVs have functionally analogous efficiency to MSCs [182] and more smoothly traffic through the BBB [183]. EVs contain numerous therapeutic elements, including cytokines, growth factors, and miRNAs [184]. MSC-EV therapy markedly contributes to cerebrovascular barrier integrity following ischemia by restoring components of BBB [184]. However, the most noticeable disadvantage of applying EVs in vivo is the limited BBB overcome after intravenous transplantation. Novel directions promoting MSC-EV delivery to definite brain regions have been investigated, such as magnetic navigation and MSC modification [184,185,186]. Exosome-mimetic nanovesicles (NVs) were generated by a serial extrusion process of stem cells, which either maintain the natural properties of EVs or attain large-scale production [187]. To advance targeting capacity, Kim and colleagues fabricated magnetic exosome-mimetic nanovesicles (MNVs) by extrusion of iron oxide nanoparticle (IONP)-containing MSCs. IONPs mediate their magnetic navigation to promote NVs migration toward the injured site and stimulate the secretion of growth factors of MSCs. Under magnetic navigation, MNV accumulation in infarct areas remarkably increased, thus inducing polarization of microglia toward the M2 phenotype and neurogenesis [185].

NVs can be inserted into many types of resident cells, such as astrocytes, pericytes, BMVECs, and macrophages/microglia, which might be the underlying mechanisms of EV-based benefits [184,185,186]. Furthermore, exosomes derived from BBB components are also potential directions, especially when the interrelationships between astrocytes, pericytes, and BMVECs are increasingly elucidated. Astrocyte-derived exosomes, whose main components include miR-190b [188], miR-361 [189], and miR-34c [190], are involved in suppressing the apoptotic process and enhancing autophagy, consequently providing a remarkable protective effect on the injured brain. In addition, pericyte-extracted exosomes could protect the blood–spinal cord barrier from the impact of inflammatory response and apoptosis post-injury through HIF-1a, Bax, Aquaporin-4, and MMP2 downregulation and TJs and Bcl-2 upgradation involved in the PTEN/AKT pathway [191]. The main components of endothelial cell-derived exosomes contain several types of miRNAs, such as miR-27a, miR-19a, miR-298, miR-195, and miR-126 [192], play a critical role in axonal proliferation and improve behavioral outcomes, whereas exosomes from endothelial progenitor cells mainly involve angiogenic promotion [193].

Engineering MSCs can secrete EVs with beneficial cargo through viral vectors or the CRISPR-Cas9 system [194,195]. Owing to the stealth features of EVs and stable gene expression of adeno-associated virus (AAV), investigators have developed a new notion of EVs known as vexosomes [194]. Based on the advantages of evasion of the body’s immune response to AAV and long-term stable gene expression, vexosomes might become a promising non-invasive approach, effectively enabling targeting to the injured brain [196]. Recently, a novel hybrid biomaterial has been studied, named enveloped protein nanocages (EPNs) or self-assembling protein nanocages, which act as potential carriers in biomedicine. EPNs in combination with vesicular stomatitis viral glycoprotein can merge with target cells and transfer cargo from one cell to another [197]. In addition, the CRISPR-Cas9 technique is a gene-editing tool with high precision and specificity. The transgenic efficiency of the CRISPR-Cas9 system is similar to that of the lentivirus vector [198]. Indeed, employing CRISPR-Cas9 to knockout (KO) toll-like receptor 4 (TLR4) gene of hMSCs significantly ameliorated the inflammatory response and secretion of EVs compared with the unedited group [199]. However, unstable gene integration restricted the efficacy of this approach and should be further improved in future studies [195]. Collectively, EV-based therapy with modifications to enhance the ability to target lesions is a feasible and highly applicable research direction in the future.

## 6. Conclusions

Following ischemia, BBB disruption initiates a series of adverse events causing vasogenic edema, neuroinflammatory response, and cell death, resulting in long-term sequelae. Recent investigations of how MSC therapy preserves ischemia-induced BBB breakdown may open up novel therapeutic targets for treating cerebrovascular diseases. The underlying mechanisms of BBB preservation include prevention of immune cells recruitment, regulation of metalloproteinases, stabilization of morphology, and crosstalk of the cellular components of the BBB. However, these mechanisms are highly complex, and interrelated signaling pathways require further investigation. To improve the therapeutic effect of MSC-based therapy, strategies to increase the overcome barrier ability of MSCs and EVs are attracting research attention, promising to provide many potential solutions in the future.

## Data Availability

Not applicable.
